# Editorial: Chemical Biology Tools for Peptide and Protein Research

**DOI:** 10.3389/fchem.2022.861699

**Published:** 2022-02-18

**Authors:** Yu-Hsuan Tsai, Hideo Iwaï, Klaus Pors

**Affiliations:** ^1^ Institute of Molecular Physiology, Shenzhen Bay Laboratory, Shenzhen, China; ^2^ Institute of Biotechnology, University of Helsinki, Helsinki, Finland; ^3^ Institute of Cancer Therapeutics, University of Bradford, Bradford, United Kingdom

**Keywords:** solid-phase peptide synthesis, non-canonical amino acid, bioorthogonal chemistry, genetic code expansion, site-specific modification, protein engineering, chemical biology

## Introduction

Peptides and proteins are important biological molecules for all living systems. The human body uses different peptides as hormones for signal transduction, whereas proteins are indispensable for cellular structure and function. Indeed, peptides and proteins are closely associated with nearly all human diseases. Not surprisingly, many peptides and proteins have been utilized for disease prevention or treatment. However, of the more than 10,000 different proteins that a human cell can produce, many are not yet fully understood (Wilhelm et al., 2014). Even for peptides and proteins that have been studied, developing them as research tools or clinical agents still faces different obstacles.

The need to address the limitations in peptide and protein research has led to the development of chemical biology tools in several aspects. Solid-phase peptide synthesis (Jaradat, 2018) is particularly useful for the production of peptides with less than 50 amino acid residues. The combination of solid-phase peptide synthesis and chemical ligation methods provides access to longer peptides and proteins without size limitation. Key advantages of these chemical approaches are the capability to include non-canonical amino acids containing diverse functional groups, as well as the production of homogeneous materials, whereas peptides and proteins isolated from cells can exist in a mixture of isoforms with different side-chain or backbone modifications.

Nevertheless, genetic means enable production of the desired proteins in large scale with ease as well as investigation of the target proteins in their native cellular environments. In addition, genetic means allow engineering of proteins with different properties, such as allosteric site for functional regulation or recognition of new substrates. Particularly, advances in analytical techniques facilitate rapid identification of protein mutants with the desired properties, supporting the development of protein engineering.

The ability to site-specifically modify proteins has also opened many opportunities for biological and medical applications. This can be achieved through bioorthogonal reactions with genetically incorporated non-canonical amino acids (de la Torre and Chin, 2021) or enzyme-mediated ligations (Xu et al., 2018). Fluorescent labeling of the target protein allows determination of its subcellular localization. Conjugation of recombinant proteins with targeting motifs may afford a target-specific delivery system. Enzyme-mediated ligation is especially useful for making backbone cyclized proteins, which often have superior stability to their linear counterparts (Hayes et al., 2021).

Below, we discuss the nine original articles and one review within this series in three categories: chemical synthesis of peptides and proteins, genetic means for protein engineering, and applications of site-specific protein modification.

## Chemical Synthesis of Peptides and Proteins

Chemical synthesis of peptides and proteins is not limited to the reservoir of canonical amino acids and enables access to their derivatives, thus facilitating elucidation of the structure-activity relationship. Towards the synthesis of human selenoprotein F (SelF), which is believed to play important roles in the quality control of the endoplasmic reticulum, Liao and He optimized the synthesis of a SelF homolog through a three-segment two-ligation strategy. Improvement in the synthetic strategy not only enabled the production of multi-milligrams of the folded synthetic protein but also set the stage for the synthesis of the native selenoprotein, of which the exact physiological functions remain largely elusive. An additional outcome of the study was the elucidation of the disulfide pairing mode of SelF. The data provided useful structural insights into the previously unresolved UGGT-binding domain of SelF.

On the other hand, Wu and Chu presented a neat synthetic route to a macrocyclic depsipeptide, pagoamide A, and evaluated its antimicrobial activity. Pagoamide A is a marine natural product, and the authors achieved its total synthesis by incorporating two thiazole building blocks in the solid-phase peptide synthesis. While the antimicrobial activity of pagoamide A was moderate against *Bacillus subtilis*, the synthetic approach was modular and is theoretically applicable to synthesizing molecules with common structural features found in peptide natural products, among them heterocycles, N-methylations, and backbone macrocyclization.

Lastly, Miret-Casals et al. employed solid-phase peptide synthesis to study furan-mediated chemical crosslinking. Although furan has been used as a warhead for chemical crosslinking, its crosslinking partners had not been experimentally characterized in detail. The authors synthesized a series of α-helical peptides, which can form coiled-coil peptide dimers. They then used these peptides to explore furan reactivity and its site-specific crosslinking. Activated furan warheads were shown to react with lysine, cysteine, and tyrosine. This *in vitro* validation demonstrates the versatility of the furan crosslinkers and provides further ground for exploiting furan-technology in developing furan-modified ligands/proteins for covalent tethering to target proteins through various amino acid side chains.

## Genetic Means for Protein Engineering

Mutagenesis is a common genetic approach to engineer proteins with novel properties. Using mutagenesis, Senoo et al. engineered metabotropic glutamate receptor 1 (mGlu1) with a novel allosteric metal-binding site. Specifically, they identified mGlu1 mutants, of which activity can be artificially induced by a protein conformational change in response to a palladium complex, Cu^2+^ or Zn^2+^. Notably, the activation of the mutants was mutually orthogonal, resulting in cell-type selective activation demonstrated in differentially transfected HEK293 cells. This direct activation *via* a coordination-based chemogenetic approach allows cell-specific activation of the target receptor, providing a useful way for investigating the physiological function of a protein in different cells.

Mutagenesis also forms the basis of directed evolution, where a protein can be transformed to equip a completely different property, such as novel enzymes that catalyze reactions invented by synthetic chemists (Arnold, 2019). A key challenge in directed evolution is to identify the desired mutants from a pool of millions or more variants. Fu et al. reviewed recent advances on sorting methods of high-throughput droplet-based microfluidics in enzyme-directed evolution. They highlighted successful cases, as well as discussed the advantages and challenges of different sorting methods.

## Applications of Site-Specific Protein Modification

Site-specific modification is a powerful strategy for studying the structure and function of peptides and proteins (Tamura and Hamachi, 2019). To study proteins within their natural environment, various bioorthogonal reactions have been developed. Since canonical amino acids lack bioorthogonal functionalities, a bioorthogonal group must be introduced into the target protein to enable site-specific modifications. This can be readily achieved through genetic code expansion, where a non-canonical amino acid can be co-translationally introduced into the desired position of the target protein (de la Torre and Chin, 2021).

In this Research Topic, Meineke et al. expanded the scope of genetic code expansion to incorporate a copper-chelating amino acid, picolyl azide lysine (PazK). They demonstrated efficient incorporation of PazK into proteins in mammalian cells, enabling improvement in copper-catalyzed azide–alkyne cycloaddition and live-cell fluorescent labeling. Results of this work can be combined with their previous finding for simultaneous dual or multiple bioorthogonal labeling on live mammalian cells.

In addition to copper-catalyzed azide–alkyne cycloaddition, azides can also react with strained alkenes and alkynes bioorthogonally. Using genetic code expansion, Johnson et al. generated two monomeric proteins containing either a *p*-azidophenylalanine (AzF) or a cyclooctyne lysine residue and demonstrated the formation of heterodimers by the two proteins. On the other hand, Wang et al. identified a variant of *Methanosarcina mazei* pyrrolysyl-tRNA synthetase for efficient incorporation of AzF. This synthetase variant enabled a generation of a model protein containing up to ten AzF residues in *Escherichia coli*. Using this pyrrolysyl-tRNA synthetase variant, they produced AzF-containing protein nanocages composed of a HER2 receptor recognition peptide and human heavy chain ferritin. Conjugation of the protein nanocages with doxorubicin by strain-promoted azide-alkyne cycloaddition afforded a cell-specific delivery system, which was demonstrated using Her2^+^ breast cancer cells and showed prolonged drug release.

Besides bioorthogonal chemistry, enzyme-mediated ligations are also useful for site-specific protein modification. Among different enzymes, asparaginyl endopeptidases (AEPs) have attracted much attention in recent years due to their superior kinetics. However, a major drawback in most enzyme-catalyzed reactions is the need of large excess of a reacting partner to drive the reaction toward completion. This is due to the reversibility and/or hydrolytic nature of the enzymes. Based on their prior knowledge of AEPs, Chen et al. engineered an AEP from *Viola canadensis* so that the enzyme variant mainly functions as a ligase for amide bond formation instead of a protease for cleavage. They achieved this by mutating residues in the non-conserved substrate-binding pockets of the enzyme. The authors also demonstrated the use of this AEP variant in generating a backbone cyclized protein as well as fluorescent labeling on live MCF7 cells.

Head-to-tail backbone cyclization of proteins and peptides has been shown to greatly enhance protein and peptide stability. By taking advantage of enzyme-mediated ligation, Hsu et al. took one step further to generate a backbone cyclized knotted protein that cannot be untied. Over a thousand knotted proteins possessing loose peptide ends (i.e., mathematically linear polypeptide chains) have been identified in nature. A mathematical knotted protein without the loose ends has opened the possibility of investigating the effect of the protein backbone topology. It is generally not possible to control the topology of the unfolded states of knotted proteins with loose ends, posing challenges in their characterization. The authors addressed this problem by performing head-to-tail cyclization of a model knotted protein. In this way, they studied the deeply trefoil-knotted YibK from *Pseudomonas aeruginosa* and investigated the effect of topological knotting in conformational entropy and protein folding.

## Perspectives

This collection of articles showcases different chemical biology tools for peptide and protein research, including solid-phase peptide synthesis, protein engineering, genetic code expansion, bioorthogonal chemistry, and enzyme-mediated ligations. Close collaboration and interplay between chemists and biologists will further expand the scope and applications of these tools. Indeed, the outcomes of this interdisciplinary field are facilitating and will certainly accelerate biological and medical research.
